# Effects of Seawater on Mechanical Performance of Composite Sandwich Structures: A Machine Learning Framework

**DOI:** 10.3390/ma17112549

**Published:** 2024-05-25

**Authors:** Norman Osa-uwagboe, Amadi Gabriel Udu, Vadim V. Silberschmidt, Konstantinos P. Baxevanakis, Emrah Demirci

**Affiliations:** 1 Wolfson School of Mechanical, Electrical, and Manufacturing Engineering, Loughborough University, Loughborough LE11 3TU, UK; n.osa-uwagboe@lboro.ac.uk (N.O.-u.); k.baxevanakis@lboro.ac.uk (K.P.B.); e.demirci@lboro.ac.uk (E.D.); 2 Air Force Research and Development Centre, Nigerian Air Force Base, Kaduna PMB 2104, Nigeria; 3 School of Engineering, University of Leicester, Leicester LE1 7RH, UK

**Keywords:** composite sandwich, machine learning, acoustic emission, damage prediction, seawater exposure

## Abstract

Sandwich structures made with fibre-reinforced plastics are commonly used in maritime vessels thanks to their high strength-to-weight ratios, corrosion resistance, and buoyancy. Understanding their mechanical performance after moisture uptake and the implications of moisture uptake for their structural integrity and safety within out-of-plane loading regimes is vital for material optimisation. The use of modern methods such as acoustic emission (AE) and machine learning (ML) could provide effective techniques for the assessment of mechanical behaviour and structural health monitoring. In this study, the AE features obtained from quasi-static indentation tests on sandwich structures made from E-glass fibre face sheets with polyvinyl chloride foam cores were employed. Time- and frequency-domain features were then used to capture the relevant information and patterns within the AE data. A *k*-means++ algorithm was utilized for clustering analysis, providing insights into the principal damage modes of the studied structures. Three ensemble learning algorithms were employed to develop a damage-prediction model for samples exposed and unexposed to seawater and were loaded with indenters of different geometries. The developed models effectively identified all damage modes for the various indenter geometries under different loading conditions with accuracy scores between 86.4 and 95.9%. This illustrates the significant potential of ML for the prediction of damage evolution in composite structures for marine applications.

## 1. Introduction

Fibre-reinforced plastic sandwich structures (FRPSS) are a common form of composite materials with growing application in various industrial sectors thanks to their superior mechanical properties, particularly their stiffness-to-weight ratio, which surpasses that of metals [1,2,3]. In the past few decades, structures made from FRPSS have been employed in the automotive, aerospace, and marine industries. Specifically, significant parts of underwater vessels, boats, and wind turbines are fabricated using FRPSS thanks to their low weight, high strength, the ease of manufacturing complete shapes, cost effectiveness, corrosion resistance, and buoyancy [4,5,6]. Composite sandwich structures typically comprise two (top and bottom) face sheets, joined together with a lightweight core. This design ensures the fabrication of a structure with relatively low weight yet sufficient stiffness and strength to withstand the in-service loads when compared to its constituent materials [7]. The overall mechanical performance of FRPSS depends on the properties and thickness of the face sheets, core, and the effectiveness of the bonds between the constituents [8,9]. It has been established that glass-fibre reinforcements are generally hydrophobic (do not absorb moisture); however, water ingress can affect the polymetric matrix resulting in plasticisation, swelling, and weakening of the interfacial bonding of the structure, thereby causing degradation of the structure’s mechanical properties [10,11,12]. Consequently, common damage modes for FRPSS subjected to seawater exposure are matrix cracking, face sheet buckling, delamination, and disbonding between the face sheet and the core [13,14]. Historically, investigations into these damage modes mostly relied on experiments and numerical simulations, which can be both time consuming and resource intensive. Non-destructive techniques such as acoustic emission (AE) have been employed in several studies to characterize the damage-resistance properties of composite sandwich structures. The results from these studies showed that AE could identify the major damage modes of the structures and correlate them with the mechanical properties of the samples [15,16,17].

In recent years, the use of machine learning (ML) algorithms to evaluate the mechanical properties and performances of composite structures under diverse loading and environmental conditions has attracted increasing attention. For instance, transfer learning, a subset of ML, shows the potential to adapt to environmental and operational variabilities, thereby accurately identifying these properties [18,19,20]. This trend is attributed to the accuracy of the models and their robustness across several applications. For example, four distinct ML methods—namely, decision tree, support vector regression (SVG), Gaussian process regression (GPR), and an ensemble method—were used to predict the damage behaviour of carbon- and glass-fibre-reinforced composites under tensile loading regimes [21]. The developed models were assessed using performance matrices such as mean absolute error (MAE), mean squared error (MSE), root mean square error (RMSE), and the coefficient of determination (R^2^). The findings indicated that GPR outperformed the other models with the highest R^2^ of 0.98 and the lowest error values [22]. Also, a convolutional neural network based on SqueezeNet was employed, using wavelet scalograms to characterize the mechanical properties of FRP composite structures [23]. The obtained results yielded a performance of more than 85% for three of the four clustered data. A deep learning approach based on the Inception Time model was proposed in [24] for damage classification of AE time- and frequency-domain features to identify fibre breakage, matrix cracking, and delamination damage modes under tension. Additionally, damage prediction models for FRP composite structures under compression [25,26], impact [27,28], and fatigue [29] were also developed using ML approaches. While demonstrations of the application of ML techniques in damage prediction and structural health monitoring abound in the literature, there is a need to further develop them for sandwich structures experiencing a combination of quasi-static localized loads and environmental conditions. Such advancements would offer valuable possibilities for material optimization and significantly enhance their practicality, particularly for offshore applications. Moreover, ML approaches have the potential to offer designers a suitable methodology with a robust dataset that can be tailored for industry-specific applications. Consequently, such models could, therefore, be beneficial for a wide range of applications.

In this study, the damage evolution in sandwich structures exposed to seawater conditions was investigated under quasi-static indentation (QSI) using the AE technique for damage characterization. The primary objective of this research is to enhance our understanding of damage in FRPSS by establishing a predictive methodology. This methodology is based on multiple ML classification algorithms, with the identification of the best-performing models determined through assessments of accuracy, recall, precision, and F1-score. In the study, time- and frequency-domain features are extracted, while the *k*-means ML algorithm is employed to identify various damage conditions. Thereafter, ensemble-based algorithms are used to predict the damage conditions of control and seawater-exposed samples. The damage modes of these two types of samples, that have different cores and are subjected to localized loading from three indenter geometries, are chosen as response variables, while the AE time- and frequency-domain features based on a rigorous feature selection process are selected as predictor variables. This is based on the direct correlation between the damage to the material, its susceptibility to moisture ingress, and the variation in the AE features during loading. Although four damage modes were identified in a previous study and were shown to be influenced by specific indenter geometries [30], the novelty of this work is its analysis of the effects of seawater on the structural health of FRPSS.

## 2. Materials, Experiment, and Methodology

### 2.1. Materials and Specimens

**Figure 1:**
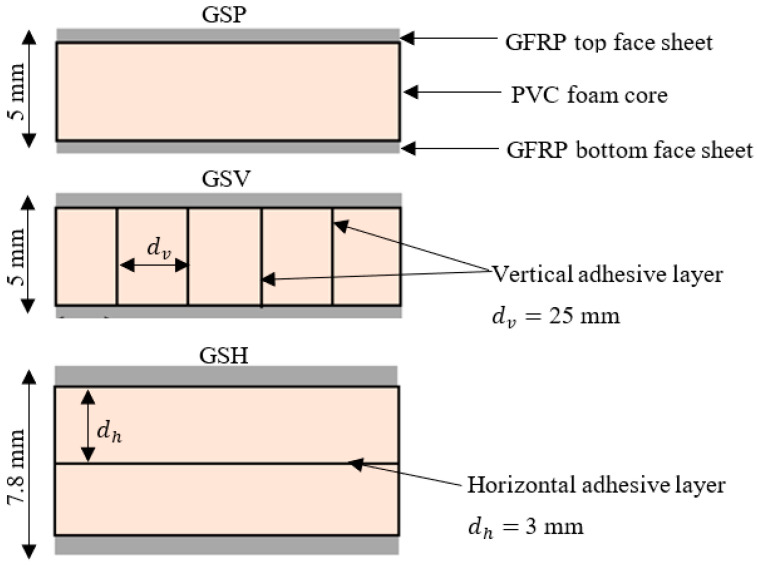
FRPSS fabrication configurations.

Face sheets of the studied FRPSS were fabricated from E-glass plain-weave fabric with a weight of 160 g/m^2^, sourced from Samson Composites Ltd. (Shenzhen, China). The matrix was epoxy resin obtained from EPOCHEM Ltd. (Lagos, Nigeria), with a volumetric ratio of 2:1. As for the core material, EASYCell 75 closed-cell PVC foam composites from Easycomposites Ltd. (Stoke-on-Trent, UK), were employed. The samples were fabricated at room temperature (27 °C) and 48% humidity in Nigeria with a curing time of 18 h. The process involved hand lay-up and vacuum-bagging techniques, resulting in sample plates with dimensions of 300 mm × 300 mm. The samples had three different core configurations, distinguished by the presence or otherwise and positions of additional adhesive layers connecting parts of the foam core (Figure 1). This fabrication method was chosen for its cost-effectiveness, simplicity, and versatility. Detailed mechanical parameters of the constituents and sample designations can be found in Table 1 and Table 2, respectively.

### 2.2. Sea Water Exposure and Moisture Absorption

**Figure 2:**
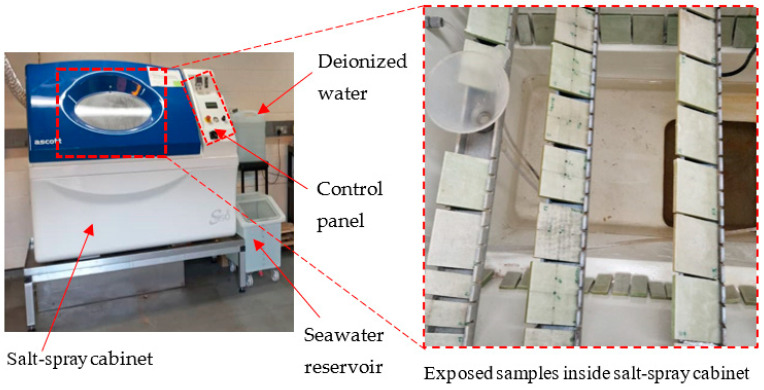
Experimental setup for salt fog spray.

To analyse the environmental effect, some manufactured samples were placed inside an Ascott S450 salt spray chamber (Figure 2), Ascott Analytical Equipment Ltd., Tamworth, UK, with salt spray at a salinity of 3.5%, following ASTM B117-19 [31] standards. Both the saturation and cabinet temperatures of the chamber were adjusted to 40 °C. This temperature setting was specifically selected as it below the glass transition temperature of the materials, thereby preventing any temperature-induced damage during the exposure.

**Figure 3:**
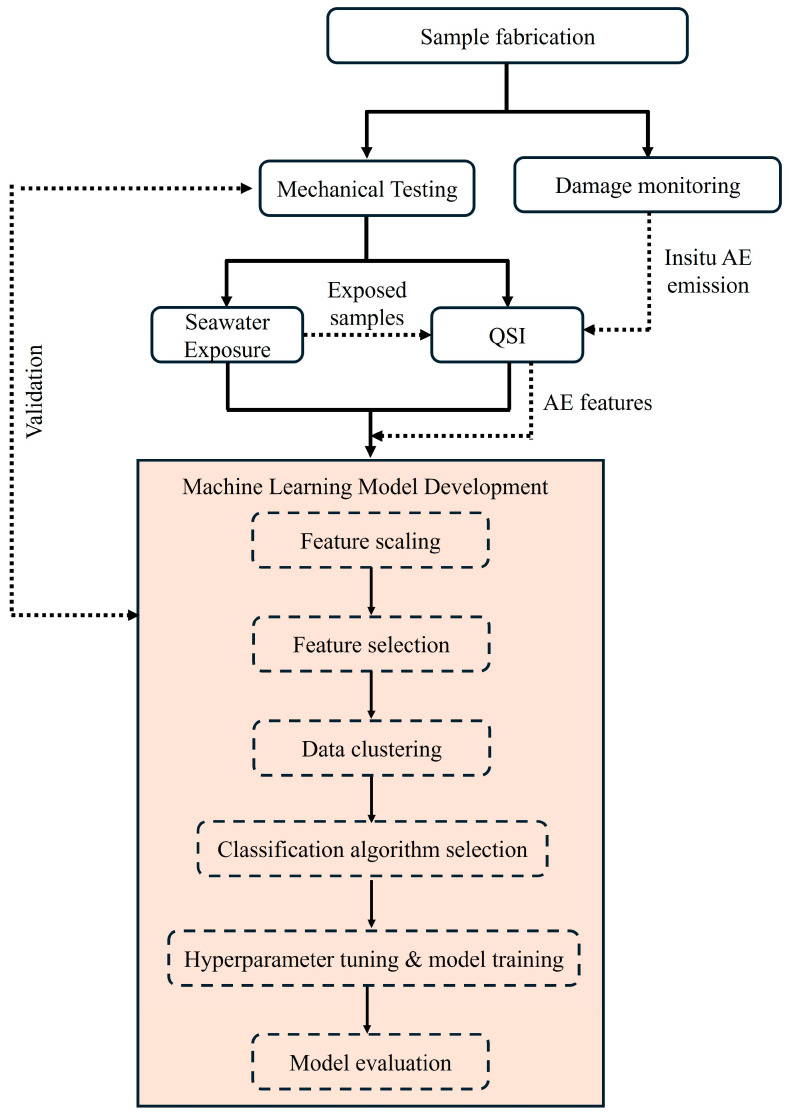
Schematic of research methodology.

Similarly, maintaining a constant pH level was crucial to prevent chemical reactions with the samples. Moisture absorption in composite structures occurs through three mechanisms: (i) moisture ingress into manufacturing-induced defects [25]; (ii) capillary/wicking along the fibre/matrix interface; (iii) combination of water molecules and hydrophilic resin groups [30]. Gravimetric measurements were conducted to determine the moisture gain in the exposed samples [15]. The average moisture uptake ($M_{t}$) increases with immersion time as defined below:

(1) $${M_{t}}_{\;}=\;\frac{M_{1}-M_{0}}{M_{0}}\times 100\%$$

where $M_{t}$ is the percentage of moisture gained, while $M_{0}$ is the initial (dry) mass of the sample and $M_{1}$ is the mass of the wet sample at a specific time. The water uptake adheres to Fickian law, i.e., it is a function of the square root of immersion time [15]. It is important to highlight that, for this study, the impact of edge corrections on moisture absorption was presumed to be minimal. This is because during fabrication, the aspect ratio of the samples was within acceptable limits (>4.5) and emphasis was placed on the meso- and macro-scale damage morphologies. A schematic of the research framework is shown in Figure 3.

### 2.3. Quasi-Static Indentation Tests

**Figure 4:**
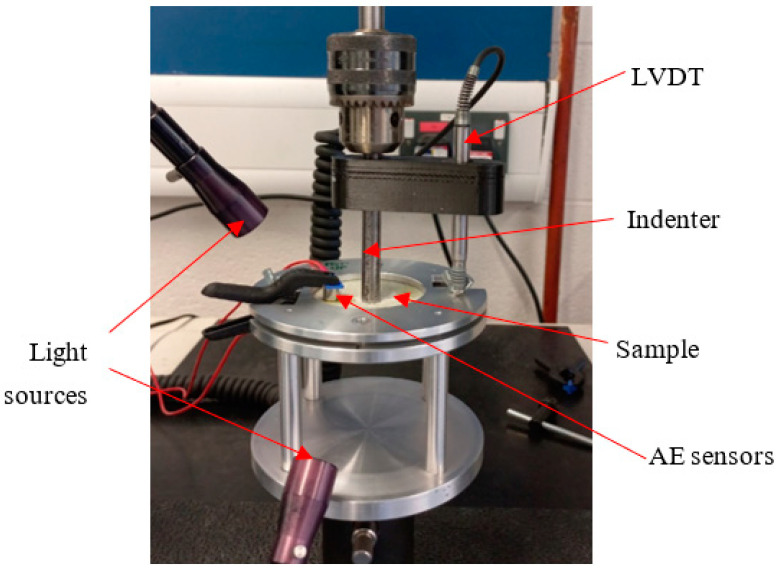
QSI experimental setup.

**Figure 5:**
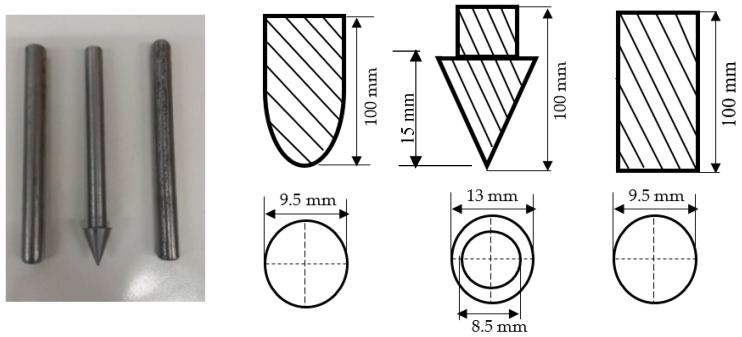
Indenter types with their dimensions.

Quasi-static indentation (QSI) tests were conducted using various indenter shapes with a minimum of 5 samples per configuration to assess the materials’ damage tolerance and provide insights into the sequence of damage. The experiments followed the ASTM D6264/D6264M-17 standard, employing a displacement control of 1 mm/min, while the vertical displacement was measured with a linear variable differential transformer (LVDT) [25]. An Instron 3369 universal testing machine with a 50 kN load cell (Instron Corporation, Norwood, MA, USA) was used for the tests, with a fixture constructed from steel plates (Figure 4). Three different indenter types made from stainless steel—flat (diameter of 9.5 mm), hemispherical (diameter of 9.5 mm), and conical (shaft diameter of 8.5 mm)—were used (Figure 5). These indenters were chosen to investigate the indentation effects of blunt, semi-blunt, and sharp foreign objects, respectively, on the FRPSS specimens. During the test, the indenter was aligned with the centre of the specimen with an offset of no more than 0.01 mm and was then applied until complete perforation of the sample. Further details regarding the QSI methodology employed can be found in our previous study [30].

### 2.4. Acoustic Emission

Several AE signal parameters, encompassing both time- and frequency-domain features, were analysed in this study. These parameters include Time (s), Class ID, Channel, Parametric, Risetime, Counts to Peak, Counts, Energy (J), Duration (s), Amplitude (dBae), ASL, Threshold, Average Frequency (Hz), RMS, Signal Strength, Absolute Energy (J), Frequency Centroid, and Peak Frequency (Hz). To capture these parameters, a structural health monitoring system, Micro-SHM, Physical Acoustics Corporation, West Windsor Township, NJ, USA), with a frequency band of 1 kHz–1 MHz equipped with 4 AE channels and 2 parametric channels was employed. However, only two channels connected to the Nano-30 AE sensors (125 kHz–750 MHz), mounted on the top and bottom face sheets (see Figure 4) were utilized.

### 2.5. ML Approach

The ML approach includes critical steps that contribute to the development of predictive models. These steps include feature scaling and selection, data clustering, classification algorithm selection, hyperparameter tuning, model training, and performance evaluation. Each of these components is essential in enhancing the accuracy, reliability, and robustness of the predictive models developed with the ML process; they are briefly discussed below.

#### 2.5.1. Feature Scaling

Feature scaling is performed on a dataset to prevent dominance issues and reduce calculation complexities. The process also helps to mitigate the impact of outliers, while enhancing the convergence and compatibility of ML algorithms. Accordingly, the time- and frequency-domain features (i.e., *X* = [*x*_1_, …, *x*_N_]^T^ ∈ R*^n^*^×*m*^) were transformed into a range between 0 and 1, and scaled as follows:

(2) $${X_{scaled}}_{\;}=\;\frac{X-X_{min}}{{X_{max}-X}_{min}}$$

where $X_{min}\;$ and $X_{max}$ are the minimum and maximum values of the features in the dataset, respectively, while $X_{scaled}$ denotes the scaled feature value after normalization.

#### 2.5.2. Feature Selection

Before model training is undertaken, it is important to identify the relevant information features. Accordingly, a feature selection process was carried out by choosing a subset of features or variables from a larger set within a dataset. The process is governed by criteria such as the relevance of the features to the response variable, the predictive strength, or the capacity of such features to enhance model performance. The primary goal of feature selection is to reduce the dimensionality of the input features and tackle the influence of highly correlated ones, to reduce the computational cost, and in some cases to enhance the efficiency and efficacy of prediction algorithms. In this work, the permutation feature importance (PFI) method was adopted for the feature selection process, which is provided with the ML classifiers, thereby making them computationally efficient and robust to outliers and noisy features [32,33]. PFI measures the contribution of each feature to the statistical performance of a model for a given dataset by randomly shuffling the values of a single feature and examining the consequent decrease in the performance score. By disrupting the connection between the feature and the value to be predicted, PFI assesses the extent to which the model depends on that specific feature. The main steps of the PFI approach are summarized below.

For instance, if *M* is a fitted predictive model and *X* a feature matrix with *n* samples and *m* features, then $X\;{\in R}^{n\times m}$, and *y* is the target vector corresponding to *n* samples, such that $y\;{\in R}^{n}$.

For each feature *j* in *X*, *y*;

For each repetition *k* in 1, …, *K*;

Randomly shuffle *j* of *X, y* to generate a corrupted version of $X,y={\tilde{X}}_{k,j},y$;

Compute reference score, $s_{k,j}$ of *M* on ${\tilde{X}}_{k,j},y$;

Compute importance $i_{j}$ for *j* defined as:

(3) $$i_{j}=s-\frac{1}{K}\sum_{k=1}^{K}s_{k,j}$$

PFI overcomes limitations of the impurity-based feature importance since it does not have a bias towards high-cardinality features and can be computed on a held-out validation set [34].

#### 2.5.3. Data Clustering

The *k*-means++ algorithm is a widely recognized unsupervised ML method for tackling clustering problems. In this work, the algorithm was employed to identify the dominant damage mechanisms. Given *X* = [*x*_1_, …, *x*_N_] with *d*-dimensional Euclidean space $R^{d}$, *A* = [*a*_1_, …, *a*_c_] is the *c* cluster centres, and $z={\left[Z_{ik}\right]}_{nxc}$, where $Z_{ik}\in \;\left\{0,\;1\right\}\;$ indicates if datapoint $x_{i}$ belongs to the *k*th cluster, *k =* 1, …, *c.* Therefore, the objective function is:

(4) $$J\left(z,A\right)=\sum_{i=1}^{n}\sum_{k=1}^{c}Z_{ik}{\left\|x_{i}-a_{k}\right\|}^{2}\;$$

such that the *k*-means++ algorithm iteratively optimizes *J*(*z*, *A*) by updating cluster centres and memberships according to specific conditions, such as

$$a_{k}=\frac{\sum_{i=1}^{n}Z_{ik}x_{ij}}{\sum_{i=1}^{n}Z_{ik}}\;\mathrm{and}$$

$$Z_{ik}=\left\{\begin{array}{cc} 1 & if{\left\|x_{i}-a_{k}\right\|}^{2}=\underset{1\leq k\leq c}{\min}{\left\|x_{i}-a_{k}\right\|}^{2}\\ 0 & otherwise \end{array}\right.$$

The challenge of *k*-means++ is usually the need to specify the number of clusters a priori [23,35,36]. It is noteworthy that the optimum clusters for the *k*-means++ were identified in the previous work [30] using two cluster-validity indices, namely the normalized Calinski–Harabasz index and the Davies–Bouldin index.

#### 2.5.4. Classification Algorithm Selection

**Figure 6:**
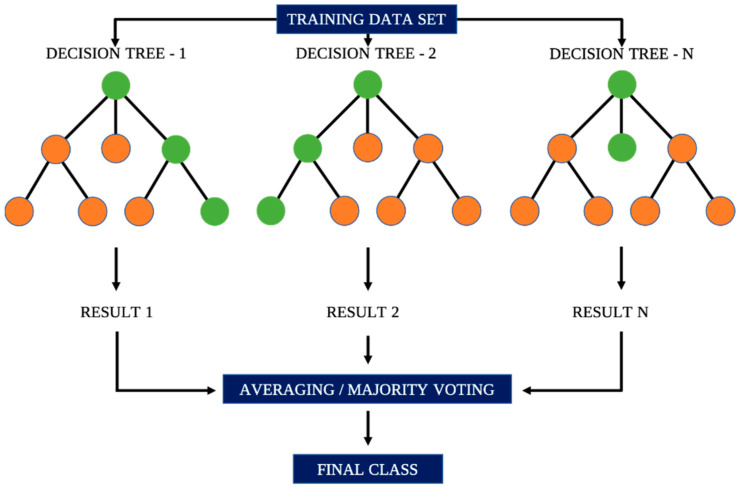
Illustration of classification using RF.

Various ML classification algorithms are available to develop prediction models. These algorithms, however, outclass each other based on the peculiarity of the dataset and prediction intentions. The Lazy Predict method was selected in this study to facilitate the selection of appropriate ML classifiers for the development of the prediction model. The method is designed for the efficient training and evaluation of multiple ML models by employing default configurations and hyperparameters for the models. This method enabled the identification and prediction of damage mechanisms by deploying a total of thirty ML classification algorithms, with the top-performing classifiers across the 9 configurations (3 × unexposed and 3 × exposed samples subjected to 3 different indenter types) selected for subsequent model development. Ensemble learning algorithms featured among the top-performing classifiers using the Lazy Predict method. These algorithms leverage multiple weak learners to make a resilient predictor. They also address overfitting and handle complex data interactions more effectively, while exhibiting resilience to noise and outliers. Additionally, they excel in scalability and efficiency when dealing with large datasets, as obtained in this study, making them suitable for the development of prediction models in diverse ML tasks. Considering the imbalanced distribution of damage-mode class data, the robustness of ensemble learners helps to prevent the majority class from dominating the learning process, allowing the model to give more attention to the minority class. This could offer ensemble learners, such as light gradient boosting machine (LightGBM), random forest (RF), and extreme gradient boosting (XGBoost), significant advantages over other algorithms in class imbalance problems [37,38,39]. RF is an ensemble learning classifier, which produces results by aggregating predictions from numerous decision trees, thereby augmenting prediction accuracy through averaging. An illustration of RF is depicted in Figure 6.

#### 2.5.5. Hyperparameter Tuning and Model Training

**Figure 7:**
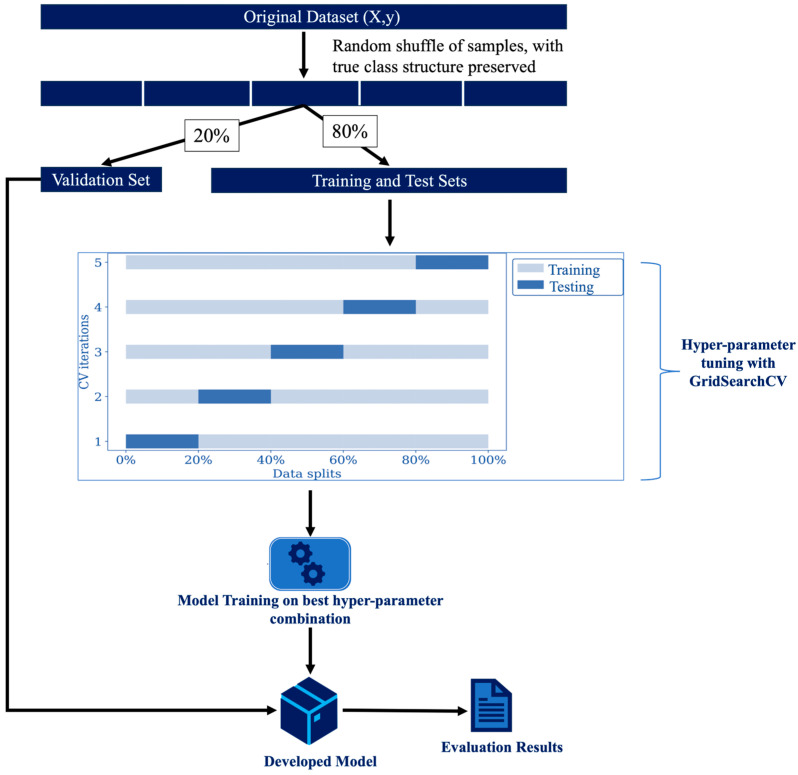
Model development process based on one hyperparameter combination.

Following the selection of the appropriate ML classifiers, a tuning process for the respective hyperparameters of the classifier is necessary to obtain the optimal set of hyperparameters to enhance the predictive performance of the model on unseen data. They can also be employed to deal with overfitting and control the computational cost of model training. Typical hyperparameters in the featured classifiers include the number of estimators, the maximum depth of the trees and the number of features to be considered in the quest for an optimal fit [40]. In this work, the GridSearch cross-validation (CV) [34] was utilized for hyperparameter tuning. It involves creating a grid of all possible hyperparameter combinations and partitioning the dataset into multiple *k*-folds/subsets (for instance, *k* = 5), where *k* − 1 folds are used to train the data and the rest are used to evaluate the model. Subsequently, the folds are rotated so that all folds are featured in the model training and testing processes. Hence, the model performance is the average mean accuracy score for each of the hyperparameter combinations, with the combination that delivers the optimal performance score adopted for the final model training on a held-out set. The model development process and the hyperparameter tuning phase for one hyperparameter combination are presented in Figure 7. To reduce the risk of bias in the developed model, a random shuffle of the data is undertaken with the true class structure preserved. Shuffling also prevents certain patterns in the original ordered data (such as timestamps) from being learned, thereby forcing the model to learn more generalized patterns rather than specific patterns related to the order of data points. Only 80% of the samples for each condition were used for the hyperparameter tuning and model training process, while the remaining 20% were used to evaluate/validate the performance of the prediction model [41].

#### 2.5.6. Model Evaluation

In assessing the performance of predictive models in detecting the damage conditions of the dataset, four machine learning metrics, namely accuracy, precision, recall, and F1-score [42], were employed. These four metrics were used because they provide more comprehensive evaluation of the classifier’s performance [43,44]. Accuracy measures the proportion of correct predictions out of the total cases, while precision and recall help us to understand how well the model performs for individual classes. Precision signifies the accuracy of positive predictions and recall indicates the model’s ability to correctly identify positive instances. F1-score is the harmonic mean of precision and recall, with both metrics contributing equally to the score. Our multidimensional approach ensures a more nuanced understanding of the classifier’s effectiveness across various scenarios and classes. The employed metrics are expressed as follows:

(5) $$\mathrm{Accuracy}=\frac{TP+TN}{TP+TN+FP+FN}$$

(6) $$\mathrm{Precision}=\frac{TP}{TP+FP}$$

(7) $$\mathrm{Recall}=\frac{TP}{TP+FN}$$

(8) $$F1\text{-}\mathrm{score}=\frac{2\cdot TP}{2\cdot TP+FP+FN}$$

where True Positives (TP) and True Negatives (TN) indicate the correct predictions by the model for positive and negative classes, respectively. Also, False Positives (FP) and False Negatives (FN) represent the instances where the model’s predictions were incorrect for positive and negative classes, respectively. The values for these metrics range between 0 and 1, with values close to unity indicating better predictive performance of the model.

## 3. Results and Discussion

### 3.1. Moisture Uptake

**Figure 8:**
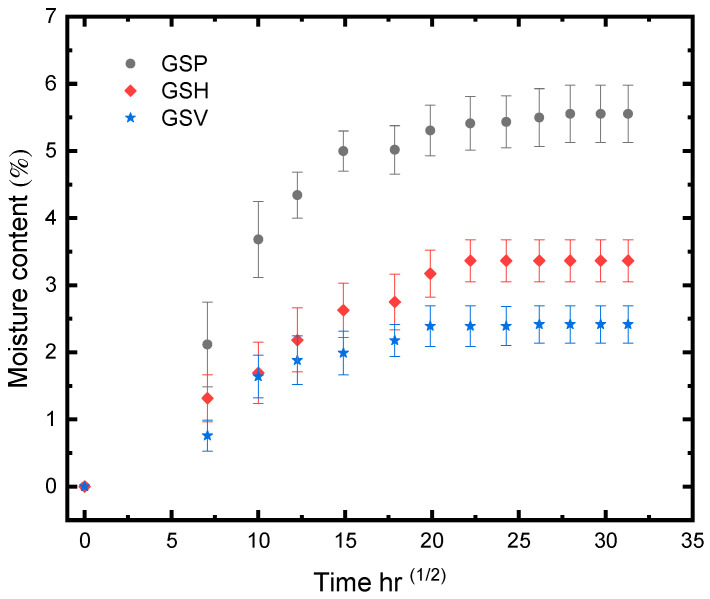
Moisture uptake of FRPSS.

The moisture absorption curve obtained from the tests adhered to Fickian law and exhibited a significant increase in the initial period of exposure (Figure 8). The FRPSS without additional adhesive layers (GSP) demonstrated a higher amount of moisture uptake than their GSV and GSH counterparts. This difference might have arisen from the lack of obstruction to the diffusion process once plasticisation of the matrix commenced, contrasting with the other samples that required more epoxy plasticisation over the exposure period. Additionally, the reduced moisture absorption observed in the GSH samples indicates that the water ingress primarily occurred in the in-plane direction, with a limited through-thickness effect (edge effect). As can be seen for all the samples, the moisture uptake continued until the saturation stage was reached, which is represented by the plateau in the curves. It should be noted that this stage was reached for GSP after a longer period of exposure while GSV and GSH attained saturation almost simultaneously. This shows that the samples with adhesive cores had similar moisture absorption properties due to the effects of the epoxy layers.

### 3.2. Quasi-Static Out-of-Plane Behaviour

#### 3.2.1. Failure Load

**Figure 9:**
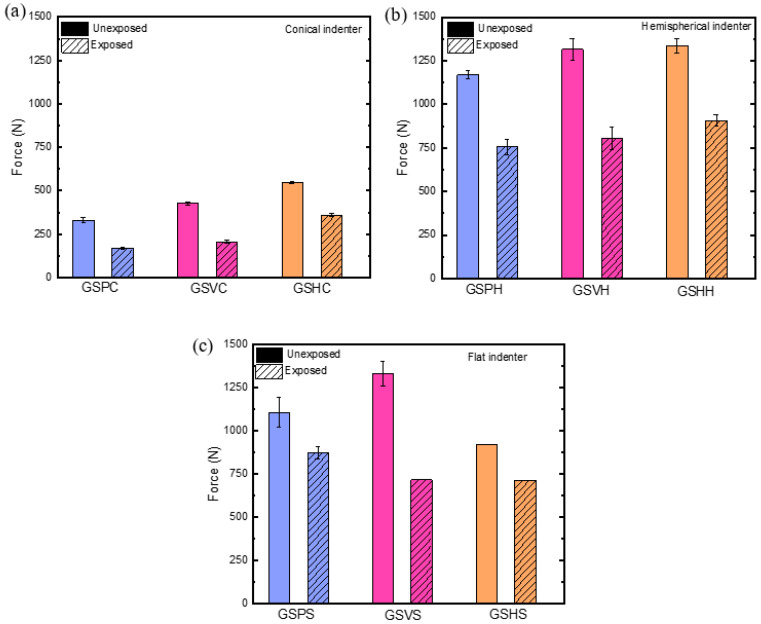
Failure load of FRPSS caused by different indenters: (**a**) conical; (**b**) hemispherical; (**c**) flat.

The mechanical performance of the FRPSS depended on both environmental exposure and indenter geometry (Figure 9). Overall, it was evident that samples subjected to the conical indenter demonstrated the lowest failure loads when compared to those subjected to flat and hemispherical indenters across all of the sample types. This phenomenon could be attributed to an early onset of localized damage at the point of contact caused by the sharp indenter, which resulted in enhanced matrix shear cracking and fibre fracture. Conversely, in cases with larger contact areas (as for hemispherical and flat indenters), more of the reinforcements came into contact with the indenter, thus enhancing the damage resistance of the loaded structure and indicating a higher load-bearing capacity until the point of sudden failure when the shear strength was exceeded. It is noteworthy that this behaviour varied with the core configuration, as samples with adhesive layers (both vertical and horizontal) exhibited greater levels of damage resistance, with the horizontal adhesive-layered samples displaying a more brittle failure character.

After the seawater exposure, the test results indicated a general decline in the load-bearing capacity for all the tested samples. Specifically, samples subjected to conical indenters exhibited the highest decrease in the failure load, with reductions of 48.9%, 51.5%, and 34.1% for the GSP, GSV, and GSH specimens, respectively. This decline can be attributed to a combination of factors, including the sharp nose of the indenter and the ageing of the structure due to seawater exposure. The conical indenter geometry led to an early onset of damage, while the seawater induced plasticisation and swelling of the epoxy matrix, affecting the adhesive bonding between the constituents of the FRPSS. At the microscale, this deterioration was characterized by a weaker fibre/matrix interface, as well as reduced intra- and interlaminar bonding, and debonding between the face sheet and the core at the macroscale [30]. In the case of indentation with larger contact areas, such as with hemispherical and flat indenters, the GSV samples were the most affected by the seawater exposure, experiencing decreases in the failure load of 38.8% and 46.1%, respectively. This could be attributed to the overall weakening of bond strength at the intersection between the top face sheet, the core, and the vertical adhesive layer, as well as the low compressive strength of the epoxy layer. Interestingly, the GSH samples performed slightly better, with a decrease of 32.1% (compared to 35.4%) for GSP under the hemispherical indenters, while there was a negligible difference for the flat indenters. This highlights that, while the core configuration could lead to enhanced damage resistance for FRPSS, it could also have significantly adverse effects for maritime applications.

#### 3.2.2. Energy Absorption Capability

Energy absorption serves as a crucial metric in comprehending the damage behaviour of composites. Hence, a comparison of the energy absorption properties of the samples under different indenter configurations can be based on the total absorbed energy $E_{a}$ obtained by integration of the area under the force–displacement curve:

(9) $$E_{a}=\int_{x_{0}}^{x_{1}}F\left(x\right)dx\;$$

**Figure 10:**
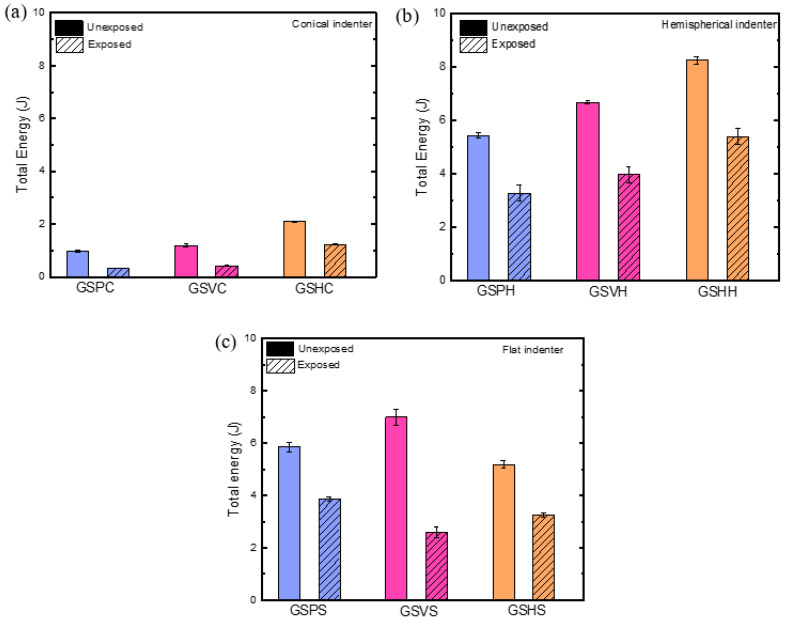
Total absorbed energy for FRPSS under different indenters: (**a**) conical; (**b**) hemispherical; (**c**) flat.

Like the trend for the failure load, it is noticeable that $E_{a}$ decreased after seawater exposure for all of the samples (Figure 10). Furthermore, specimens loaded with the conical indenter (GSPC, GSVC and GSHC) exhibited the lowest energy absorption capability and experienced the largest decline after the seawater exposure, with reductions of 66.9%, 65.2%, and 41.4%, respectively. As previously explained, this could be attributed to the easier penetration of the sharp nose and accelerated degradation due to seawater ageing. The energy performance of specimens subjected to hemispherical and flat indenters varied in a way similar to the failure load trend.

### 3.3. AE Results

**Figure 11:**
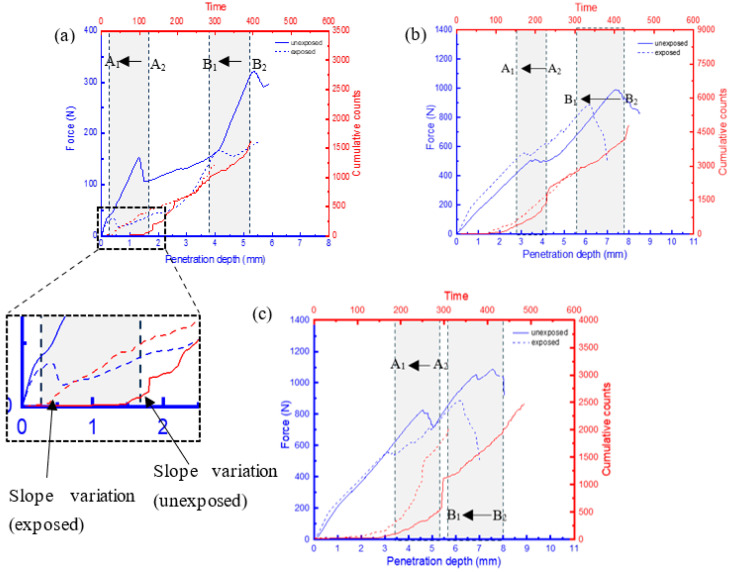
Relationship between load and AE cumulative counts of GSP specimen with different indenters: (**a**) conical; (**b**) hemispherical; (**c**) flat.

**Figure 12:**
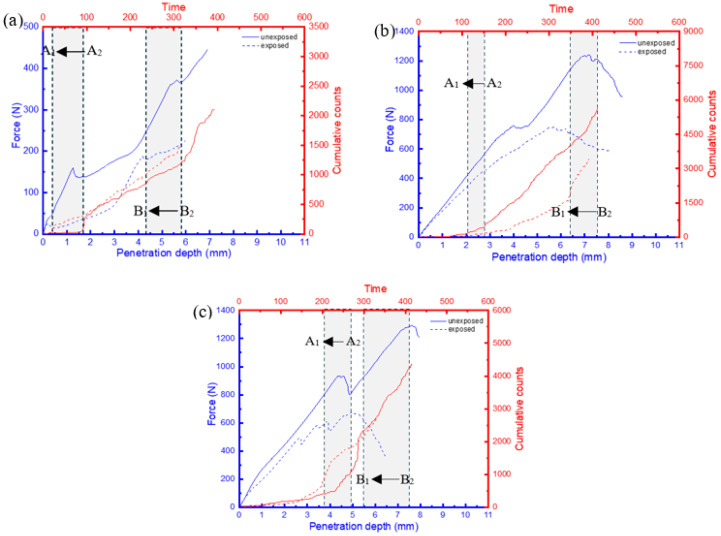
Relationship between load and AE cumulative counts for GSV specimen with different indenters: (**a**) conical; (**b**) hemispherical; (**c**) flat.

**Figure 13:**
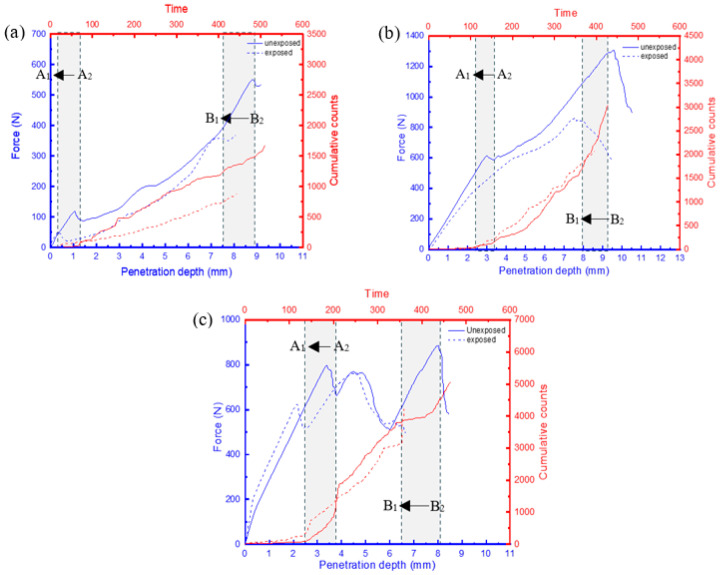
Relationship between load and AE cumulative counts for GSH specimen with different indenters: (**a**) conical; (**b**) hemispherical; (**c**) flat.

It has been shown that the behaviour of AE features, such as cumulative counts, can offer crucial insights into assessing the damage mechanisms and failure characteristics of composite materials. This is due to their ability to facilitate a comprehensive classification of distinct damage zones under quasi-static loading conditions [21,22,23]. These distinct zones can be identified when there is a change in the gradient of the cumulative count’s slope (Figure 11—zoomed-in section), indicating a transition in the load-bearing capacity of the sample characterized by the presence of a damage sequence [22]. Considering that the displacement speed for all samples was kept constant, the time change would provide an insight into the effects of moisture uptake on the rate of damage at critical loads. A comparison of the force–displacement plots and the AE cumulative counts for the FRPSS samples, both unexposed and exposed to seawater, is depicted in Figure 11, Figure 12 and Figure 13.

There was a reduction in the time taken for the change in the cumulative count slopes of the samples after seawater exposure to occur for penetrations of the top and bottom face sheets. This time difference can be calculated by $A_{2}-A_{1}\;$ for the top face sheet penetration and $B_{2}-B_{1}$ for the bottom face sheet penetration. Here $A_{2}\;and\;B_{2}$ represent the penetration time for the top face sheet for the unexposed specimen while $A_{1}\;and\;B_{1}$ denote the penetration time for the exposed samples, respectively. GSP samples subjected to conical, hemispherical, and flat indenters (Figure 11) experienced drops of 80%, 75%, and 16% in the top face sheet and 28%, 28%, and 27% in the bottom one, respectively. For GSV, the values were 78%, 27%, and 26% for the top face sheet and 25%, 17%, and 27% for the bottom face sheet, respectively, (Figure 12) while the respective values for GSH were 73%, 31%, and 29% and 15%, 15%, and 20% (Figure 13). It can be seen that the conical indenters caused the largest drop in the time in the top face sheet for all samples which reinforces the heightened damage onset of sharp indenters on exposed samples. Interestingly, the time interval for complete failure ranged from 15% to 28% as a result of the varying combination of the damage resistances of the core, indenter shape, and friction in the through-thickness direction. Consequently, it can be concluded that the time taken for the onset of damage for FRPSS after seawater exposure to the top face sheet was primarily driven by the indenter geometry, while the other constituents played a contributory role in the subsequent damage sequence.

### 3.4. ML Setup

The primary tools utilized in the ML part of this study were the Python programming language along with the *pandas*, *NumPy*, *joblib*, *scikit-learn (sklearn)*, and *Matplotlib* libraries. These libraries facilitated essential tasks that were necessary for model development, such as data manipulation, numerical computation, the implementation of ML algorithms, and visualisation. Owing to the large dataset size, *joblib* aided in parallelising the execution capabilities, thereby reducing the cost of computationally expensive tasks such as cross-validation and hyperparameter tuning. These computations were carried out using the ALICE high-performance computing facility at the University of Leicester, UK. For each specimen type (e.g., GSPH), the experimental data for both control and exposed samples were uploaded and combined into one data frame, and the normalization process was applied using the Min–Max scaler, transforming each value of each feature to be within the range of 0 to 1. The resulting dataset was randomly shuffled using a predefined random seed and split in ratio of 4:1. Thus, 80% of the data were used for model training, with while the remaining 20% were used for evaluating the model performance. During the splitting process, the data were stratified based on the response class labels, so that the samples of all class labels were equally distributed to alleviate issues arising from class imbalance during the model training process. Thereafter, a clustering analysis was initiated based on the *k*-means++ algorithm using the amplitude and peak frequency features. The number of clusters to identify as well as the number of centroids to generate was set at *n* = 4 in our previous study [30].

For computations, a predefined random state was used to allow for reproducibility. The resulting predicted clusters served as the response variable. Thus, representing the damage modes for the respective specimens. Consequently, the Lazy Predict algorithm was initialized to assess the model performance of thirty ML classification algorithms on their default configurations and hyperparameters for the models. For each specimen, the dataset was reshuffled based on the NumPy random state generator, with Lazy Predict providing the preliminary assessment for model performance. The average accuracy results from five iterations led to the identification of the three top-performing classifiers across the eight damage modes that were predicted (four each for exposed and unexposed sample). These included LightGBM, RF, and XGBoost. Hyperparameter tuning was subsequently undertaken with GridSearch CV to determine the best hyperparameter combination that offered the best generalisation performance on the respective classifiers. In the tuning process, *k* = 5 cross-validation folds were selected with an accuracy set as the baseline scoring parameter. PFI was then carried out by shuffling the features 10 times, and the model refitted to estimate the importance of the feature based on the mean decrease in accuracy. The features were then sorted based on their importance and stacked into a two-dimensional array, in which only the highest ranked feature was in the first row and all the features in the fifteenth row. Using a joblib parallelization, each of the rows of features was fitted on their respective classifiers, and the row which delivered the best model performance was evaluated on the initially held-out validation set based on the four performance metrics. It is noteworthy that the weighted averages were specified for the parameters of the precision, recall, and F1-score matrices, which accounted for the class imbalance of the samples. Finally, the confusion matrices of the best-performing model of the three classifiers were computed and discussed in the subsequent sections.

### 3.5. Feature Analysis

**Figure 14:**
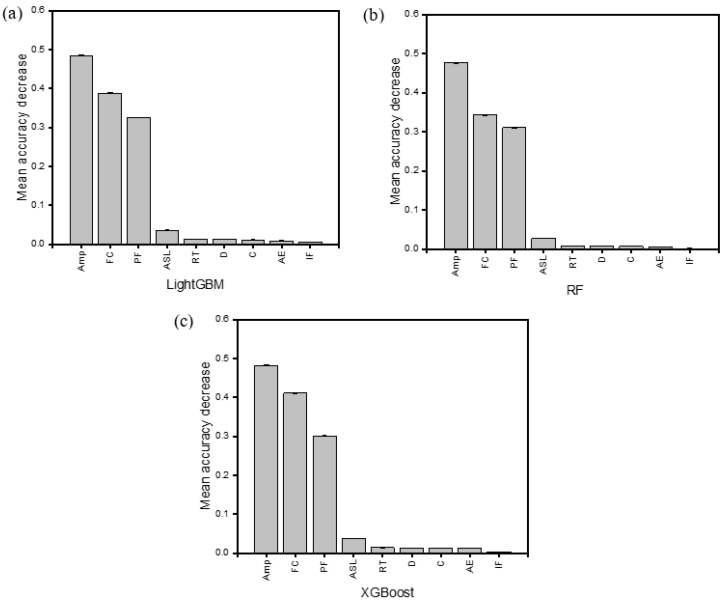
Sample feature analysis of GSVH with best performing algorithm: (**a**) LightGBM; (**b**) RF; (**c**) XGBoost (Amp—amplitude; FC—frequency centroid; PF—peak frequency; ASL—average signal level; RT—rise time; D—duration; C—counts; AE—absolute energy; IF—initiation frequency).

Generally, the obtained results indicated that the amplitude, frequency centroid, and peak frequency features were the major signals contributing to the accuracy of the predictive ML models (Figure 14) for all samples. Furthermore, the frequency centroid was more dominant among them, as shown in Table 3.

Using Table 3 (in which the highest values of AE features for each sample are highlighted), an analysis of the mean accuracy decrease (MAD) value for GSPC AE features shows that the removal of the AE amplitude feature from the dataset decreased the mean accuracy by 42%. Furthermore, it can also be observed that, in the LightGBM and XGBoost models, the FC was the most important feature, contributing to about 45–70% of the predictive power.

For specimens loaded with the hemispherical indenter, the PF contributed significantly to about 30–50% of the MAD. On the contrary, it was observed that, for the flat and conical indenters, peak frequency had a mostly insignificant influence on the predictive power of the models. This can be linked to the material’s behaviour at the transition points (elastic to plastic) underneath these indenters. The hemispherical indenter offers a more consistent transition gradient at the critical load points than the conical and flat indenters, which caused an abrupt failure at these critical points, thereby creating a larger scatter of data points. A similar occurrence for the ML regression algorithm was reported in [45,46]. Therefore, for composite structures loaded with conical and flat indenters, the peak frequency cannot be relied upon alone to assess the damage evolution using AE signals. In practical terms, structural health monitoring of composite materials for marine applications with AE could be achieved for similar sharp and blunt incident objects using the frequency centroid and amplitude. For semi-blunt objects, peak frequency inputs could also provide valuable data for failure monitoring. For the models built on the RF classifier, the amplitude, frequency centroid, and peak frequency were the main contributors to the MAD. This could indicate a higher level of robustness to outliers of RF in damage prediction for data sets with varying levels of fluctuation. Generally, the threshold feature did not contribute to the MAD, implying its irrelevance in the development of a predictive model for damage assessment based on ensemble learning algorithms. Since energy had a negligible value in the MAD, it was not included in the analysis.

#### 3.5.1. Hyperparameter Tunning Results

The hyperparameters, range, selected parameters, and mean cross-validation scores of GSVC that were used to identify the damage modes are presented in Table 4. Other hyperparameters not shown in the table were used in their default states.

Following this, the models were trained using the selected hyperparameter values (which provided an optimal performance while dealing with overfitting) for the respective classifier attributes to identify the damage sequence.

#### 3.5.2. Identification of Damage Sequence

**Figure 15:**
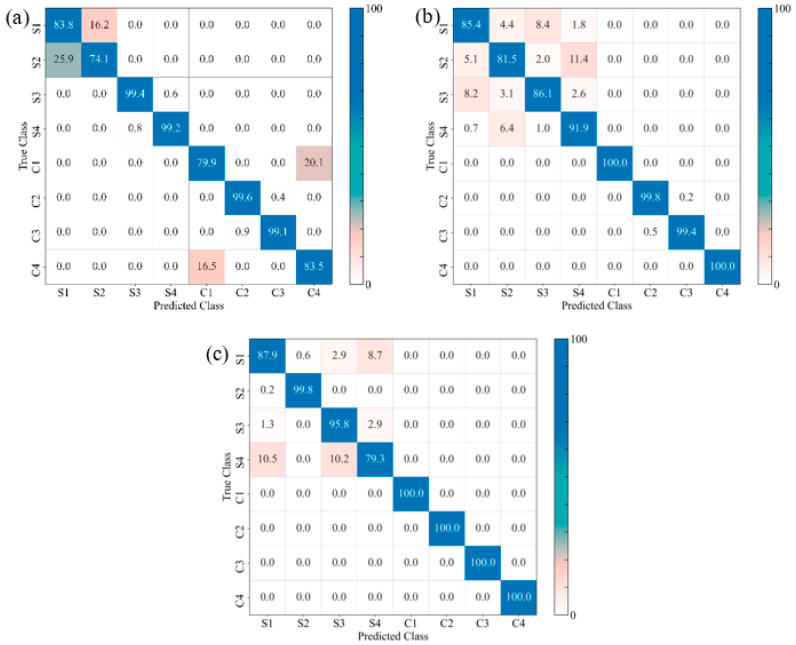
Confusion matrices for different FRPSS: (**a**) GSPC; (**b**) GSPH; (**c**) GSPS.

**Figure 16:**
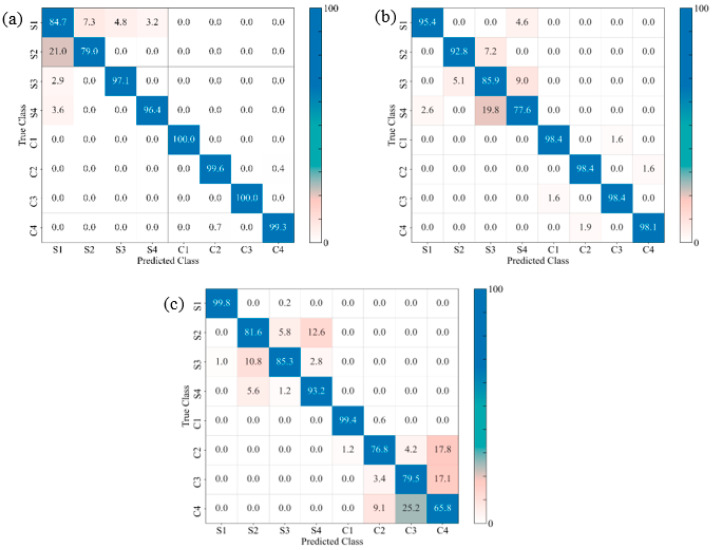
Confusion matrices for different FRPSS: (**a**) GSVC; (**b**) GSVH; (**c**) GSVS.

**Figure 17:**
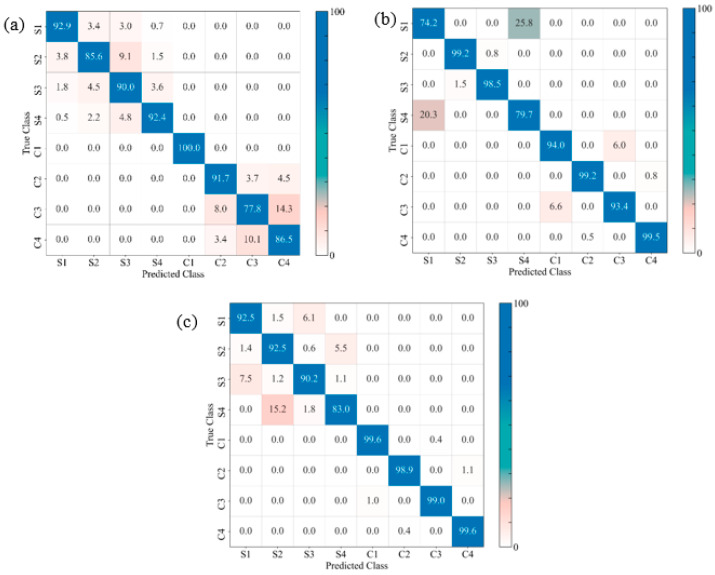
Confusion matrices for different FRPSS: (**a**) GSHC; (**b**) GSHH; (**c**) GSHS.

The confusion matrices of the prediction models for the GSP, GSV, and GSH samples allow us to visualize their performance by comparing the true and the predicted labels (Figure 15, Figure 16 and Figure 17, respectively). For the sake of analytical comparisons and interpretability, the individual quantitative values were normalized, demonstrating the results of clustering (C1, C2, C3, C4, S1, S2, S3 and S4), with C1–C4 and S1–S4 representing the four damage modes (matrix cracking, delamination, fibre breakage, and core damage) for the unexposed and exposed samples, respectively. The identification of the damage modes for FRPSS was discussed in previous studies [27]. Generally, the developed models could clearly distinguish between unexposed and exposed specimens, with the control samples exhibiting a higher extent of correct predictions between 66 and 100%. This is because, as demonstrated previously, seawater exposure led to the degradation of the mechanical performance of the constituents of the FRPSS and the entire structures. This degradation caused a faster onset of damage in the matrix due to plasticisation, as well as a more pronounced weakening at the constituent’s interface (matrix–fibre interface, intra-laminate/interlaminate, and face sheet/core). It can be observed that the correct classifications of the GSP samples loaded with the hemispherical and flat indenters had higher values than those loaded with the conical one, with the lowest being 74.1% and 79.9% for GSPC-S2 and GSPC-C1, respectively (Figure 15a). For the GSV samples, the lowest classification results were GSVH-S4 and GSVS-C4 at 77.6% (Figure 16b) and 65.8% (Figure 16c), respectively. Lastly, GSHC-C3 and GSHH-S1 showed the lowest values of 77.8% (Figure 17a) and 74.2% (Figure 17b) for the GSH samples, respectively. These results demonstrate the ability of the developed models to deal with damage induced by moisture uptake and the dominant influence of seawater in the prediction capabilities of the ML algorithm. A description of the class clustering data for exposed and unexposed samples is given in Table 5.

In terms of the average performances for the eight damage modes, C1–C4 and S1–S4, Table 6 shows the model performance values for each sample loaded with the various indenters with the highest values highlighted. The models with the highest performance values for each sample had a range of 86.4%–95.9% across the selected performance indicators (namely, accuracy, precision, recall, and F1-score). Although the four metrics yielded similar scores in terms of their predictive capabilities on each sample, Friedman tests taken at the level of α = 0.05 resulted in F-statistic and *p*-values of 8.181 and 0.043 for LightGBM; 9.243 and 0.026 for RF; and 7.950 and 0.047 for the XGBoost models, respectively. The tests, therefore, indicated that there was a statistically significant difference in performance between the metrics and the models. Accordingly, at least one of the models (LightGBM, RF or XGBoost) performed differently from the others on the four evaluated metrics. Thus, the XGBoost model demonstrated the best overall performance across all the studied samples. This was noted on the GSVS sample, achieving accuracy, precision, recall, and F1-score values of 0.9587, 0.9592, 0.9587, and 0.9587, respectively. Specifically, the LightGBM model exhibited the highest performance for the GSV sample, while the XGBoost model demonstrated the highest performance for the GSH sample, across all three of the indenter types employed.

## 4. Conclusions

This study investigated the effect of moisture uptake on the damage to FRPSS with GFRP face sheets and PVC foam cores caused by loading with different indenters, which is relevant for marine applications. Multiple ML predictive models were applied to the data collected with AE during QSI tests for both control and seawater-treated samples. The results obtained from the study indicated the following:

- The decline in the load-bearing capacity for all samples after the seawater exposure was attributed to several factors. Samples loaded with the conical indenter experienced the highest decrease in the maximum load, with reductions of 48.9%, 51.5%, and 34.1% for GSP, GSV, and GSH specimens, respectively. For indentation cases with a higher contact area, the GSV samples were notably impacted by seawater exposure, with reductions of 38.8% and 46.1%, while the GSH samples showed a slightly better performance, decreasing by 32.1% (compared to 35.4% for GSP) under the hemispherical indenter and exhibiting a negligible difference under the flat indenter.
- In terms of energy absorption, a similar trend was observed, with this parameter demonstrating the largest decrease for the samples loaded with the conical indenter—66.9%, 65.2%, and 41.4% (respectively, for GSP, GSV, and GSH) after seawater exposure. This could be attributed to the easier penetration of the sharp (conical) indenter and accelerated degradation due to seawater ageing. Furthermore, the energy performance of specimens subjected to hemispherical and flat indenters varied in a way similar to the failure load trend.
- The AE amplitude, frequency centroid, and peak frequency were the major signals contributing to the accuracy of the predictive ML models for all samples, with the FC being dominant. The MAD values for GSPC, GSVC, and GSHC decreased the mean accuracy after the removal of the AE amplitude by 42%, 52%, and 69%, respectively, with LightGBM models. The FC was the most important feature, contributing 45–70% to predictive power in LightGBM and XGBoost models. For the hemispherical indenter, the PF contributed significantly (30–50% MAD). The models showed high performances (86.4–95.9%) in distinguishing between the unexposed (control) specimens and the exposed ones. The lowest correct classification rates were observed for samples loaded with the conical indenter.

Overall, the developed ML models could clearly distinguish between unexposed and exposed specimens, with the control samples exhibiting a higher extent of correct predictions between 66 and 100%. The reason for this, as shown by the experimental results, was the effect of moisture uptake on the degradation of mechanical properties of the constituents in the FRPSS. Also, it was observed that the XGBoost model performed best overall, achieving a 95.9% accuracy for the GSVS samples. This paper thus demonstrated the potential of ML techniques for damage prediction in marine structures and components and the viability of using these techniques with data from in situ AE inputs, which could be important for various industrial applications.

## Figures and Tables

**Table 1:** Mechanical properties of materials [27,29].

Material	Young’s Modulus (GPa)	Shear Modulus (GPa)	Tensile Strength (MPa)	Poisson Ratio	Density (g/cm^3^)
	*E*	*G*_12_		$\nu_{12}$	ρ
E-glass fabric	72.39	8.27	3100–3800	0.26	2.25
PVC foam	0.075	0.028	1.89	-	0.075
Epoxy matrix	3.2–3.5	-	70–80	0.29	1.16

**Table 2:** Sample nomenclature.

Designation	Description
GSPC	Glass sandwich with pure core/conical indenter
GSVC	Glass sandwich with vertical adhesive layered core/conical indenter
GSHC	Glass sandwich with horizontal adhesive layered core/conical indenter
GSPH	Glass sandwich with pure core/hemispherical indenter
GSVH	Glass sandwich with vertical adhesive layered core/hemispherical indenter
GSPS	Glass sandwich with pure core/flat indenter
GSVS	Glass sandwich with vertical adhesive layered core/flat indenter
GSHS	Glass sandwich with horizontal adhesive layered core/flat indenter

Note: Below, notation _c and _s after sample designations is used for unexposed and exposed samples, respectively.

**Table 3:** Results of AE feature analysis.

Sample	Model	AE Features								
		Amp	FC	PF	ASL	RT	D	C	AE	IF
GSPC	LightGBM	**0.42**	0.07	0.00	0.06	0.00	0.01	0.02	0.03	0.01
	RF	0.35	**0.41**	0.50	0.06	0.01	0.01	0.01	0.01	0.02
	XGBoost	0.37	**0.67**	0.00	0.03	0.04	0.03	0.02	0.01	0.01
GSPH	LightGBM	0.43	**0.46**	0.45	0.10	0.01	0.05	0.06	0.07	0.01
	RF	0.46	0.41	**0.50**	0.06	0.01	0.01	0.02	0.01	0.01
	XGBoost	0.49	0.43	**0.51**	0.08	0.01	0.02	0.02	0.03	0.01
GSPS	LightGBM	0.42	**0.70**	0.00	0.06	0.00	0.02	0.02	0.03	0.01
	RF	**0.42**	0.35	0.38	0.03	0.00	0.00	0.01	0.00	0.01
	XGBoost	0.43	**0.70**	0.00	0.05	0.00	0.02	0.02	0.04	0.01
GSVC	LightGBM	0.43	**0.59**	0.00	0.16	0.04	0.03	0.04	0.02	0.04
	RF	**0.40**	0.30	0.26	0.13	0.02	0.02	0.03	0.00	0.03
	XGBoost	0.43	**0.59**	0.00	0.16	0.04	0.04	0.04	0.03	0.04
GSVH	LightGBM	**0.48**	0.39	0.33	0.04	0.01	0.01	0.01	0.01	0.01
	RF	**0.48**	0.34	0.31	0.03	0.01	0.01	0.01	0.00	0.00
	XGBoost	0.49	0.43	**0.51**	0.08	0.01	0.02	0.02	0.03	0.01
GSVS	LightGBM	**0.44**	0.70	0.00	0.03	0.00	0.02	0.05	0.03	0.01
	RF	**0.43**	0.31	0.37	0.05	0.00	0.02	0.03	0.00	0.00
	XGBoost	0.43	**0.70**	0.00	0.07	0.00	0.03	0.05	0.03	0.00
GSHC	LightGBM	0.46	**0.69**	0.00	0.01	0.01	0.03	0.02	0.03	0.00
	RF	**0.41**	0.36	0.28	0.01	0.01	0.02	0.01	0.02	0.01
	XGBoost	0.46	**0.69**	0.00	0.01	0.01	0.03	0.02	0.03	0.01
GSHH	LightGBM	0.36	**0.47**	0.31	0.05	0.01	0.01	0.02	0.01	0.01
	RF	0.38	**0.46**	0.22	0.03	0.01	0.01	0.01	0.00	0.01
	XGBoost	0.38	**0.45**	0.32	0.05	0.01	0.02	0.02	0.02	0.01
GSHS	LightGBM	0.35	**0.63**	0.00	0.09	0.03	0.03	0.04	0.02	0.02
	RF	**0.33**	**0.33**	0.28	0.07	0.02	0.01	0.02	0.01	0.02
	XGBoost	0.34	**0.63**	0.00	0.09	0.03	0.03	0.04	0.02	0.02

**Table 4:** Hyperparameters of classifiers and their studied ranges for GSVC.

Model	Attribute	Range	Selected Value	Mean Score
LightGBM	colsample_bytree	[0.7, 0.8, 0.9, 1]	0.9	0.8836
	learning rate	[0.01, 0.1, 0.2]	0.01	
	max_depth	[−1, 5, 10, 15]	10	
	min_child_weight	[0.001, 0.01, 0.1]	0.001	
	n_estimators	[100, 200, 250, 300]	300	
RF	criterion	[‘gini’, ‘entropy’, ‘log_loss’]	‘entropy’	0.8944
	max_depth	[None, 2, 3, 4, 5, 7, 8, 10, 20]	None	
	max_features	[None, ‘sqrt’, ‘log2′]	‘sqrt’	
	min_samples_leaf	[1, 2, 3, 4, 5]	1	
	min_samples_split	[2, 4, 5, 7, 8, 10]	7	
	n_estimators	[100, 150, 200, 250, 300]	300	
XGBoost	colsample_bytree	[0.7, 0.8, 0.9, 1]	0.9	0.8955
	gamma	[0, 0.1, 0.2]	0	
	learning rate	[0.01, 0.1, 0.2]	0.01	
	max_depth	[3, 6, 9]	6	
	n_estimators	[100, 200, 300]	200	

**Table 5:** Class clustering data description.

Class Clustering Data Description								
Sample	Cluster	Value	Sample	Cluster	Value	Sample	Cluster	Value
GSP								
GSPC_c1	C1	16,381	GSPH_c1	C1	47,633	GSPS_c1	C1	45,924
GSPC_c2	C2	15,177	GSPH_c2	C2	52,810	GSPS_c2	C2	69,598
GSPC_c3	C3	32,798	GSPH_c3	C3	38,213	GSPS_c3	C3	72,779
GSPC_c4	C4	16,988	GSPH_c4	C4	54,834	GSPS_c4	C4	45,146
GSPC_s1	S1	17,142	GSPH_s1	S1	57,804	GSPS_s1	S1	26,283
GSPC_s2	S2	17,898	GSPH_s2	S2	70,847	GSPS_s2	S2	53,096
GSPC_s3	S3	18,911	GSPH_s3	S3	85,418	GSPS_s3	S3	70,230
GSPC_s4	S4	18,655	GSPH_s4	S4	88,616	GSPS_s4	S4	36,562
GSV								
GSVC_c1	C1	22,860	GSVH_c1	C1	25,789	GSVS_c1	C1	28,432
GSVC_c2	C2	21,091	GSVH_c2	C2	34,000	GSVS_c2	C2	27,548
GSVC_c3	C3	25,386	GSVH_c3	C3	21,235	GSVS_c3	C3	27,665
GSVC_c4	C4	16,755	GSVH_c4	C4	26,295	GSVS_c4	C4	23,663
GSVC_s1	S1	19,879	GSVH_s1	S1	53,640	GSVS_s1	S1	53,889
GSVC_s2	S2	18,700	GSVH_s2	S2	61,286	GSVS_s2	S2	36,701
GSVC_s3	S3	14,859	GSVH_s3	S3	51,369	GSVS_s3	S3	35,678
GSVC_s4	S4	19,492	GSVH_s4	S4	81,788	GSVS_s4	S4	61,885
GSH								
GSHC_c1	C1	20,454	GSHH_c1	C1	40,256	GSHS_c1	C1	47,697
GSHC_c2	C2	13,528	GSHH_c2	C2	31,579	GSHS_c2	C2	32,055
GSHC_c3	C3	35,224	GSHH_c3	C3	47,866	GSHS_c3	C3	17,419
GSHC_c4	C4	17,772	GSHH_c4	C4	27,235	GSHS_c4	C4	36,516
GSHC_s1	S1	26,507	GSHH_s1	S1	17,311	GSHS_s1	S1	45,513
GSHC_s2	S2	22,917	GSHH_s2	S2	59,395	GSHS_s2	S2	24,719
GSHC_s3	S3	23,774	GSHH_s3	S3	35,561	GSHS_s3	S3	33,314
GSHC_s4	S4	12,656	GSHH_s4	S4	70,114	GSHS_s4	S4	41,140

**Table 6:** Summary of best-performing ML models.

Sample	Model	Performance Indicators			
		Accuracy	Precision	Recall	F1-Score
GSPC	LightGBM	0.9103	0.9106	0.9103	0.9102
	RF	0.9106	0.9108	0.9106	0.9105
	XGBoost	**0.9111**	**0.9114**	**0.9111**	**0.9110**
GSPH	LightGBM	0.9357	0.9356	0.9357	0.9355
	RF	0.9434	0.9433	0.9434	0.9432
	XGBoost	**0.9453**	**0.9453**	**0.9453**	**0.9452**
GSPS	LightGBM	0.9562	0.9556	0.9562	0.9557
	RF	**0.9568**	**0.9563**	**0.9568**	**0.9563**
	XGBoost	0.9568	0.9562	0.9568	0.9563
GSVC	LightGBM	0.8974	0.8973	0.8974	0.8972
	RF	0.8971	0.8969	0.8971	0.8968
	XGBoost	**0.8996**	**0.8995**	**0.8996**	**0.8995**
GSVH	LightGBM	0.9429	0.9429	0.9429	0.9429
	RF	0.9421	0.9422	0.9421	0.9421
	XGBoost	**0.9436**	**0.9437**	**0.9436**	**0.9435**
GSVS	LightGBM	0.9584	0.959	0.9584	0.9583
	RF	0.9556	0.956	0.9556	0.9555
	XGBoost	**0.9587**	**0.9592**	**0.9587**	**0.9587**
GSHC	LightGBM	0.9548	0.9553	0.9548	0.9548
	RF	0.9535	0.9544	0.9535	0.9535
	XGBoost	**0.9548**	**0.9554**	**0.9548**	**0.9549**
GSHH	LightGBM	**0.9389**	**0.9394**	**0.9389**	**0.9392**
	RF	0.9384	0.9391	0.9384	0.9387
	XGBoost	0.9383	0.9388	0.9383	0.9385
GSHS	LightGBM	**0.8636**	**0.8640**	**0.8636**	**0.8630**
	RF	0.8616	0.8614	0.8616	0.8612
	XGBoost	0.8632	0.8635	0.8632	0.8627

## Data Availability

Dataset available on request from the authors.
